# Epidemiology and antimicrobial resistance trends of pathogens causing urinary tract infections in Mwanza, Tanzania: A comparative study during and after the implementation of the National Action Plan on Antimicrobial Resistance (2017-2022)

**DOI:** 10.1016/j.ijid.2024.107208

**Published:** 2024-10

**Authors:** Vitus Silago, Katarina Oravcova, Louise Matthews, Stephen E. Mshana, Heike Claus, Jeremiah Seni

**Affiliations:** 1 Department of Microbiology and Immunology, Weill Bugando School of Medicine, Catholic University of Health and Allied Sciences, Mwanza, Tanzania; 2 Institute for Hygiene and Microbiology, University of Würzburg, Würzburg, Germany; 3 Institute of Biodiversity, Animal Health and Comparative Medicine, University of Glasgow, Glasgow, UK

**Keywords:** Antimicrobial resistance (AMR) surveillance, National action plan on AMR, Urinary tract infection, Hospital levels

## Abstract

**Objectives:**

To delineate the epidemiology and antimicrobial resistance (AMR) trends of pathogens causing urinary tract infections (UTIs) during (June 2019-June 2020) and after (March-July 2023) the implementation of the National Action Plan on AMR 2017-2022 in Mwanza, Tanzania.

**Methods:**

This cross-sectional study was conducted among 2097 patients with clinical symptoms of UTIs during (n = 1144) and after (n = 953) the National Action Plan on AMR 2017-2022. Quantitative urine culture was done to isolate significant bacteria causing UTI, which were then identified to the species level and tested for antimicrobial susceptibility. Tabulations, descriptive, and logistic regression analyses were used to analyze categorical and continuous variables, as well as the association between outcome and independent variables. Statistical significance was defined as *P* ≤0.05 at a 95% confidence interval (CI).

**Results:**

The overall prevalence of culture-positive UTIs was 22.8% (479 of 2097; 95% CI: 21.1-24.7%), with no significant difference between the study periods (21.8% [249 of 1144; 95% CI: 19.5-24.3%]) vs 24.1% (230 of 953; 95% CI: 21.5-26.9%), *P* = 0.274). We observed a significant increase in resistance to ciprofloxacin (32.0% vs 45.8%, *P* = 0.0481) and third-generation cephalosporins (marked by extended-spectrum β-lactamase–producing *Enterobacterales* [ESBL-PE], 38.7% vs 56.9%, *P* = 0.0307). Additionally, UTIs caused by ESBL-PE is significantly common among patients in higher-tier hospitals (58.4% vs 34.0%; OR [95% CI]: 2.51 [1.41-4.48], *P* = 0.002).

**Conclusions:**

There was a significant increase in bacterial resistance to ciprofloxacin and third-generation cephalosporins, as well as ESBL-PE. These results emphasize the critical need to enhance AMR surveillance, improve infection prevention and control measures, and strengthen antimicrobial stewardship programs.

## Highlights

• Study on the implementation of National Action Plan on Antimicrobial Resistance.
• The largest sample size to study the epidemiology and antimicrobial resistance.
• No significant change in the prevalence of urinary tract infections.
• Change in bacteria species causing urinary tract infections between two periods.
• The trend of antimicrobial resistance is increasing significantly.

## Introduction

Antimicrobial resistance (AMR) is currently a highly significant global concern in the realm of public health [1]. Recently, the excessive and inappropriate use of antibiotics has resulted in the emergence and spread of bacteria resistant to multiple antimicrobials [1]. Consequently, managing infections, including urinary tract infections (UTIs), is now facing a substantial threat because existing antibiotic treatments have become less effective [1]. To address this issue, numerous countries, including Tanzania, are implementing their National Action Plans on AMR (NAP-AMR) adapted from the World Health Organization Global Action Plans on AMR [2,3].

UTIs are one of the most prevalent bacterial infections worldwide, for example, in 2019, more than 404.6 million people had UTIs [4]. The emergence of AMR has complicated the management of UTIs. The World Health Organization has identified 12 priority pathogens based on their AMR potentials [5]. Of these, critical priority pathogens include carbapenemase-producing *Acinetobacter baumannii* and *Pseudomonas aeruginosa* and carbapenemase and extended-spectrum β-lactamase (ESBL)–producing Enterobacterales*,* as well as the high priority pathogen, methicillin-resistant *Staphylococcus aureus* [5,6]. All these pathogens can potentially cause UTIs, underscoring an urgent need to institute AMR testing and surveillance to guide specific management. Tanzania rolled-out its NAP-AMR 2017-2022, which, among others, focuses on four objectives, i.e. creating awareness through information, education, and communication; fostering AMR research and surveillance; mitigating infection prevention and control; and promoting antimicrobial stewardship (AMS) [3]. However, AMS and AMR surveillance on UTIs and blood stream infections in Tanzania is exclusively focused on tertiary and secondary hospitals (higher-tier hospitals) and not on primary hospitals (lower-tier hospitals). Furthermore, the NAP-AMR program's roll-out was challenged by the COVID-19 pandemic, which was first declared on March 16, 2020 in Tanzania [7], resulting in a surge of antibiotics consumption used in health care facilities and the communities [8].

Therefore, this study was designed to address these critical research gaps by involving five hospitals spanning from primary (lower-tier) to secondary and tertiary (higher-tier) hospitals to delineate epidemiology and resistance trends of pathogens causing UTIs in Mwanza, Tanzania through a multisectoral and multicenter project, “Supporting the NAP-AMR” (SNAP-AMR), during the implementation of the Tanzanian NAP-AMR 2017-2022. The SNAP-AMR project collaborated with the Tanzanian Ministry of Health between June 2019 and June 2020 to support all four strategic objectives of the NAP-AMR and build laboratory capacity at lower-tier hospitals by establishing bacteriology laboratories to perform cultivation, phenotypic detection, and susceptibility testing and reporting of resistance phenotypes.

## Materials and methods

### Study design, duration, and setting

This cross-sectional hospital-based study was conducted between June 2019 and June 2020 (first cohort, during NAP-AMR) and between March and July 2023 (second cohort, after NAP-AMR) in five health care facilities in Mwanza, Tanzania. Bugando Medical Centre is a tertiary zonal referral hospital with a capacity of 1080 beds, serving a catchment of about 18.8 million people from the Lake Victoria Zone regions (Mwanza, Mara, Kagera, Kigoma, Simiyu, Shinyanga, and Geita). Sekou Toure Regional Referral Hospital, which has approximately 450 beds, is a regional referral hospital for the Mwanza Region, serving a population of 3.6 million people. Sumve Designated District Hospital (SDDH), with a bed capacity of 265, is a designated district hospital that serves the population of Kwimba district. Magu District Hospital (MDH) is a district hospital for Magu District, with an estimated bed capacity of 150. Misungwi District Hospital (MisDH) is a district hospital for Misungwi District, with a bed capacity of 128. SDDH, MDH, and MisDH serve a total population of 1.3 million people. In this study, higher-tier hospitals refer to Bugando Medical Centre and Sekou Toure Regional Referral Hospital, whereas lower-tier hospitals refer to MDH, MisDH, and SDDH.

### Population, selection criteria, and data collection

In the current study, we enrolled patients exhibiting clinical symptoms of UTIs, such as pyuria, dysuria, lower abdominal pain, flank pain, or fever, according to Tanzania's standard treatment guidelines [9]. The clinical diagnosis of UTIs was done by clinicians in the respective health care facilities. Urine samples were then collected and sent to the microbiology laboratory of the corresponding health care facilities for culture and antibiotic susceptibility testing. Those patients whose urine samples were received in the laboratory were eligible for enrollment in this study. Each patient was enrolled once. The patients’ information such as age, sex, hospital number, and ward of admission or clinic of the visit were extracted from laboratory request forms or laboratory information management systems. The information captured from the patients’ request forms or laboratory information management systems was used to track patients to collect further related information; sociodemographic and clinical data were collected using a pretested structured questionnaire. A total of 51 (4.3%) patients enrolled during NAP-AMR were excluded from the final data set before data analysis due to incomplete information. However, no patient enrolled after NAP-AMR was excluded due to incomplete information.

### Laboratory procedures

A 10-µl loop was used for quantitatively inoculation of urine sample on 5% sheep blood agar and MacConkey agar plates, followed by aerobic incubation at 35 ± 2°C for 18 to 24 hours. Bacterial growth of ≥10^5^ CFU/mL was considered significant for UTIs. The in-house biochemical identification tests were used for identification of isolated bacteria as previously documented [10]. The Kirby-Bauer disc diffusion method was used for antimicrobial susceptibility testing (AST), and the zones of inhibition were interpreted based on Clinical and Laboratory Standards Institute guidelines [11]. All culture media and antibiotic discs were manufactured by HiMedia, Maharashtra, India. All laboratories followed the same standard operating procedures to ensure the reproducibility of laboratory results across hospitals. The isolates were initially analyzed in the laboratories of the hospitals to guide the patients’ management and then transported to the Microbiology Research Laboratory of the Catholic University of Health and Allied Sciences in Mwanza, Tanzania for confirmation and storage at −80°C until further analysis.

Bacterial species and AST were verified at the Institute for Hygiene and Microbiology of the Julius Maximilians University of Würzburg, Würzburg, Germany. Species identification was done by matrix-assisted laser desorption ionization-time of flight on VITEK MS (bioMérieux, Nürtingen, Germany) and 16S rRNA Sanger sequencing, whenever necessary. To determine bacterial susceptibility to antibiotic agents, minimum inhibitory concentrations were determined by VITEK 2 (bioMérieux, Nürtingen, Germany) using the following AST cards: AST-P654 (*Staphylococcus* spp. and other gram-positive bacteria [GPB]), AST-N214 (Enterobacterales and *Acinetobacter* spp.), AST-N371 (*E. coli*), AST-P655 (*Enterococcus* spp. and *Streptococcus* spp.), and AST-N248 (*Pseudomonas* spp.). To interpret the AST results, the European Committee on Antimicrobial Susceptibility Testing clinical break points (version 13.0, 2023) were applied [12].

In the current study, coagulase-negative Staphylococci (CoNS) and other rare bacterial pathogens were considered significant pathogens causing UTIs based on the following: (i) every patient enrolled in this study showed symptoms indicative of UTIs, (ii) pure (one type of microbial growth) and significant growth (at least 10^5^ CFU/mL) on culture media, and (iii) previous studies reported that CoNS and rare bacterial pathogens possess important virulence genes/factors associated with infections, including UTIs [13,14].

### Detection of multidrug-resistance phenotypes

*S. aureus* strains showing resistance toward cefoxitin were considered as methicillin-resistant *S. aureus*, whereas ESBL-producing strains of *E. coli* and *K. pneumoniae* were detected based on simultaneous assessment of the inhibitory effects of cefepime, cefotaxime, and ceftazidime alone and in the presence of clavulanic acid [12].

### Quality control

*E. coli* ATCC 25922 and *S. aureus* ATCC 25923 were used as control organisms for the validation of laboratory protocols such as biochemical identification and antibiotic susceptibility testing.

### Data management and analysis

Microsoft Excel was used for data cleaning and coding, whereas STATA 15.0 was used for data analysis. Continuous and categorical data are presented as median (interquartile range) and proportions or percentages, respectively. Univariate and multivariate logistic regression analyses were used to determine the association between independent variables (e.g. age, sex, exposure to antibiotics, etc.) and the outcome variable (i.e. UTIs by ESBL-producing *Enterobacterales* [ESBL-PE]). Independent variables showing significant levels of association (*P* <0.05) by univariate analysis were included in the multivariate analysis. In addition, the Wilcoxon rank-sum test (Mann–Whitney *U* test) was used to compare the median age of culture-positive and -negative groups. A *P* ≤0.05 at 95% confidence interval (CI) was considered statistically significant.

## Results

### Sociodemographic and clinical characteristics of patients with the clinical diagnosis of UTI during and after NAP-AMR

A total of 2097 patients were enrolled (1144 during NAP-AMR and 953 after NAP-AMR). The median (interquartile range) age of all patients was 26 (6-42) years and patients enrolled after NAP-AMR were slightly older than those enrolled during NAP-AMR (29 [8-44] vs 23 [5-40] years). Overall, female patients accounted for 59.3% (1244 of 2097), 56.9% (651 of 1144) during NAP-AMR and 62.2% (593 of 953) after NAP-AMR. Of all patients, 39.5% (828 of 2097) had primary education level and 44.0% (923 of 2097) were self-employed (Table 1).

**Table 1:** Sociodemographic and clinical characteristics of patients with clinical diagnosis of urinary tract infections during and after the NAP-AMR.

Characteristics	Categories	Overall (N = 2097)	During NAP-AMR (N = 1144)	After NAP-AMR (N = 953)	Chi-square test
		n (%)	n (%)	n (%)	*P*-value
Median (interquartile range) age in years		26 (6-42)	23 (5-40)	29 (8-44)	<0.0001^a^
Sex	Male	853 (40.7)	493 (43.1)	360 (37.8)	0.014
	Female	1244 (59.3)	651 (56.9)	593 (62.2)	
Residency	Rural	1057 (50.4)	595 (52.0)	462 (48.5)	0.107
	Urban	1040 (49.6)	549 (48.0)	491 (51.5)	
Education level	None	700 (33.4)	462 (40.4)	238 (24.9)	<0.0001
	Primary	828 (39.5)	397 (34.7)	431 (45.2)	
	Secondary	340 (16.2)	164 (14.3)	176 (18.5)	
	Tertiary	229 (10.9)	121 (10.6)	108 (11.3)	
Occupation	None	944 (45.0)	565 (49.0)	379 (39.8)	<0.0001
	Employed	230 (11.0)	140 (12.2)	90 (9.4)	
	Self-employed	923 (44.0)	439 (38.4)	484 (50.8)	
Healthcare facility	BMC	668 (31.8)	273 (23.9)	395 (41.4)	<0.0001
	SRRH	312 (14.9)	102 (8.9)	210 (22.0)	
	MDH	428 (20.4)	280 (24.5)	148 (15.5)	
	SDDH	337 (16.1)	212 (18.5)	125 (13.1)	
	MisDH	352 (16.8)	277 (24.2)	75 (7.9)	
Level of healthcare facility	Higher-tier	980 (46.7)	375 (32.8)	605 (63.5)	<0.0001
	Lower-tier	1117 (53.2)	769 (67.2)	348 (36.5)	
Patient category	Outpatient	1411 (67.3)	667 (58.3)	744 (78.1)	<0.0001
	Inpatient	686 (32.7)	477 (41.7)	209 (21.9)	
Ward/clinic of enrollment	Medical	1433 (68.3)	618 (54.0)	815 (82.8)	<0.0001
	Pediatric	473 (22.5)	389 (34.0)	84 (8.8)	
	Surgical	107 (5.1)	88 (7.7)	19 (2.0)	
	Others^b^	110 (5.2)	49 (4.3)	61 (6.4)	
History of fever past 3 months	Yes	868 (41.4)	528 (46.1)	340 (35.7)	<0.0001
	No	1229 (58.6)	616 (53.9)	613 (64.3)	
History of admission 3 months	Yes	258 (12.3)	171 (15.0)	87 (9.1)	<0.0001
	No	1839 (87.7)	973 (85.0)	866 (90.9)	
History of antibiotic use 3 months	Yes	606 (28.9)	365 (31.9)	241 (25.3)	0.001
	No	1491 (71.1)	779 (68.1)	712 (74.7)	
Patient on antibiotic during sampling	Yes	542 (25.8)	539 (47.1)	3 (0.3)	<0.0001
	No	1555 (74.1)	605 (52.9)	950 (99.7)	
Classes of antibiotics taken by patients during the study period	Penicillins	310 (57.2)	309 (57.3)	1 (33.3)	<0.0001
	Cephalosporins	130 (23.9)	129 (23.9)	1 (33.3)	
	Quinolones	92 (16.9)	92 (17.1)	0 (0.0)	
	Aminoglycosides	67 (12.4)	65 (12.1)	2 (66.7)	
	Macrolides	23 (4.2)	23 (4.3)	0 (0.0)	
	Nitroimidazoles	49 (9.0)	47 (8.7)	2 (66.7)	
	Carbapenems	3 (0.5)	3 (0.6)	0 (0.0)	
Chronic disease	Yes	214 (10.2)	122 (10.7)	92 (9.6)	0.447
	No	1883 (89.8)	1022 (89.3)	861 (90.4)	
Types of chronic diseases	Hypertension	81 (37.8)	45 (36.9)	36 (39.1)	<0.0001
	Sickle cell disease	38 (17.7)	32 (26.2)	6 (6.5)	
	Diabetes mellitus	57 (26.6)	31 (25.4)	26 (28.3)	
	HIV	31 (14.5)	25 (20.6)	6 (6.5)	
	Cancer	19 (8.9)	0 (0.0)	19 (20.6)	
	Chronic kidney disease	1 (0.5)	1 (0.8)	0 (0.0)	

AICU, adult intensive care unit; Gyn/Obs, gynecology/obstetrics; NICU, neonatal intensive care unit; NAP-AMR, National Action Plan on Antimicrobial Resistance.

a Wilcoxon rank-sum (Mann–Whitney) test.

b During NAP-AMR: others: neonatology unit (n = 21), AICU (n = 12), NICU (n = 10), and Gyn/Obs (n = 6). After NAP-AMR: others: Gyn/Obs (n = 28), oncology (n = 14), neonatology unit (n = 9), AICU (n = 8), and NICU (n = 2).

Of 2097 patients, 1117 (53.2%) were from lower-tier hospitals and 1141 (54.4%) were enrolled as outpatients. A higher proportion of patients was enrolled as outpatients after NAP-AMR (78.1%, 744 of 953) than during NAP-AMR (58.3%, 667 of 1144). Most patients (68.3%, 1433 of 2097) were enrolled from medical wards/clinics. The rates of admission within 3 months before enrollment in the current study during and after NAP-AMR were 15.0% and 9.1%, respectively. The rates of antibiotic use within 3 months before enrollment in the current study during and after NAP-AMR were 31.9% and 25.3%, respectively. Although, the current antibiotic use on admission or visit was 47.1% during NAP-AMR and 0.3% after NAP-AMR, with penicillins (57.2%) and cephalosporins (23.9%) predominating during NAP-AMR (Table 1).

### Culture-positive UTIs in patients with the clinical diagnosis of UTIs

Of 2097 urine samples examined, 479 had significant microbial growth on culture, resulting in a cumulative prevalence of culture-positive UTIs of 22.8% (95% CI: 21.1-24.7%). The prevalence of UTIs increased slightly but not significantly, from 21.8% (249 of 1144; [95% CI: 19.5-24.3%]) during NAP-AMR to 24.1% (230 of 953; [95% CI: 21.5-26.9%]) after NAP-AMR (*P* = 0.274).

**Figure 1:**
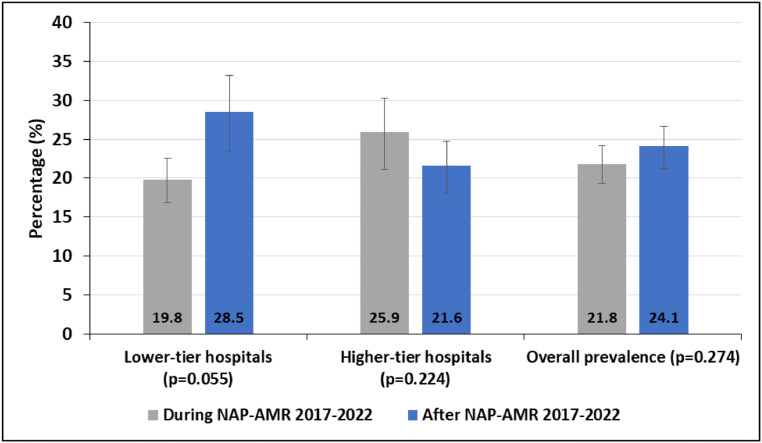
Prevalence of laboratory-confirmed urinary tract infections in patients presenting with clinical symptoms of urinary tract infections. Error bars showing 95% confidence intervals. NAP-AMR = national action plan on antimicrobial resistance.

The decrease of UTIs in higher-tier hospitals from 25.9% (97 of 375; [95% CI: 21.5-30.6%]) during NAP-AMR to 21.6% (131 of 605; [95% CI: 18.4-25.2%]) after NAP-AMR (*P* = 0.224) and the increase of UTIs in lower-tier hospitals from 19.8% (152 of 769; [95% CI: 17.0-22.8%]) during NAP-AMR to 28.5% (99 of 348; [95% CI: 23.8-33.5%]) after NAP-AMR (*P* = 0.055) were not significant (Figure 1).

### Bacterial species causing UTIs

**Figure 2:**
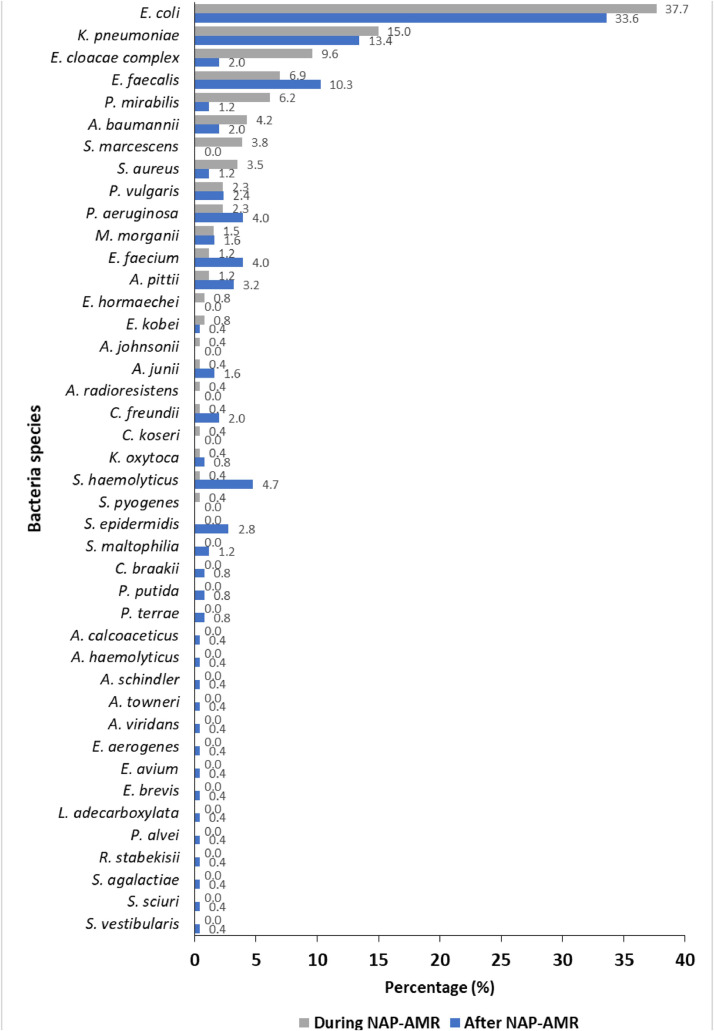
Bacteria species causing urinary tract infections during and after NAP-AMR. NAP-AMR = national action plan on antimicrobial resistance.

Of 479 positive urine culture, a total of 513 isolates were identified: 260 during NAP-AMR and 253 after NAP-AMR. Up to two different species were detected in one positive sample. Gram-negative bacteria (GNB) were most prevalent causative agents of UTIs in both periods; however, the proportion decreased significantly from 88.1% (229 of 260) during NAP-AMR to 74.3% (188 of 253) after NAP-AMR (*P* <0.0001). The species causing >50% of the cases during NAP-AMR were *E. coli* (37.7%, 98 of 260), *K. pneumoniae* (15.0%, 39 of 260), *E. cloacae* complex (9.6%, 25 of 260), *E. faecalis* (6.9%, 18 of 260), and *P. mirabilis* (6.1%, 16 of 260), whereas the species causing >50% of the cases after NAP-AMR were *E. coli* (33.6%, 85 of 253), *K. pneumoniae* (13.4%, 34 of 253), *E. faecalis* (10.3%, 26 of 253), *S. haemolyticus* (4.7%, 12 of 253), and *E. faecium* and *P. aeruginosa* (4.0%, 10 of 253 each). Further species identified during and after NAP-AMR are listed in Figure 2.

### AMR trends of GNB causing UTIs

In the comparison of AMR during and after NAP-AMR, we observed an increased percentage resistance of GNB toward the antibiotic agents tested. A significant increase was observed toward cefpodoxime (42.2-57.4%, *P* = 0.0271), ceftazidime (23.2-41.3%, *P* = 0.0249), and ciprofloxacin (32.0-45.8%, *P* = 0.0481) (Table 2).

**Table 2:** Antimicrobial resistance of gram-negative bacteria causing urinary tract infections during and after national action plan on antimicrobial resistance.

Antibiotic agent tested	% Resistance in lower-tier hospitals			% Resistance in higher-tier hospitals			Overall % resistance		
	During (N = 55-133)	After (N = 35–77)	*P*-value	During (N = 43-96)	After (N = 50-111)	*P*-value	During (N = 98-229)	After (N = 85-188)	*P*-value
	%R [95% CI]	%R [95% CI]		%R [95% CI]	%R [95% CI]		%R [95% CI]	%R [95% CI]	
AMP	83.2 [74.7-89.7]	84.5 [72.5-92.6]	0.4217	94.5 [86.5-98.5]	92.5 [85.1-96.9]	0.3092	87.8 [82.1-92.2]	89.4 [83.4-93.8]	0.3341
AMC	65.0 [48.3-79.4]	72.4 [52.8-87.3]	0.3087	79.4 [62.1-91.3]	89.6 [77.3-96.5]	0.1179	71.6 [59.9-81.5]	83.1 [72.8-90.7]	0.0678
SXT	61.9 [52.3-70.9]	64.5 [51.3-76.3]	0.3930	82.7 [72.7-90.2]	78.1 [68.5-85.9]	0.2458	70.6 [63.7-76.9]	72.8 [65.1-79.5]	0.3498
CPD	33.6 [24.8-43.4]	46.4 [32.9-60.3]	0.1539	54.8 [42.7-66.5]	64.1 [53.5-73.9]	0.1766	42.2 [34.9-49.8]	57.4 [49.1-65.5]	0.0271
CTX	25.2 [17.3-34.5]	37.9 [22.5-51.6]	0.1693	50.0 [37.9-62.0]	55.8 [45.2-65.9]	0.2952	35.2 [28.2-42.7]	49.0 [40.8-57.2]	0.0513
CAZ	17.6 [10.9-26.1]	29.5 [18.5-42.6]	0.1964	31.2 [21.1-42.7]	48.1 [38.3-58.0]	0.0838	23.2 [17.4-30.0]	41.3 [33.8-49.2]	0.0249
GEN	24.6 [16.9-33.5]	16.1 [8.0-27.6]	0.2899	46.5 [35.7-57.6]	46.7 [36.9-56.7]	0.4925	34.0 [27.5-41.0]	35.3 [28.1-43.1]	0.4390
CIP	22.8 [15.5-31.6]	36.1 [24.2-49.4]	0.1556	44.2 [33.5-55.3]	51.4 [41.5-61.3]	0.2481	32.0 [25.6-38.9]	45.8 [38.0-53.7]	0.0481
TZP	21.6 [14.4-30.4]	25.0 [15.5-36.6]	0.3979	44.0 [33.2-55.3]	32.7 [23.9-42.4]	0.1624	31.3 [24.8-38.3]	29.6 [23.0-36.9]	0.4221
TGC	7.5 [2.1-18.2]	13.2 [4.4-28.1]	0.3916	16.3 [7.3-29.6]	9.1 [3.0-19.9]	0.3560	11.7 [6.2-19.6]	10.7 [5.3-18.9]	0.4705
NIT	2.5 [0.1-13.2]	0.0 [0.0-11.9]	-	0.0 [0.0-10.3]	2.1 [0.0-11.1]	-	1.4 [0.0-7.3]	1.3 [0.0-7.0]	-
IPM	2.6 [0.5-7.5]	0.0 [0.0-5.7]	-	4.7 [1.3-11.5]	10.5 [5.3-17.9]	0.3639	3.5 [1.4-7.1]	6.6 [3.3-11.5]	0.3883
MEM	0.0 [0.0-3.2]	0.0 [0.0-5.7]	-	3.5 [0.7-11.5]	9.5 [4.7-16.8]	0.3693	1.5 [0.3-4.3]	6.0 [2.9-10.7]	0.3765

AMC, amoxicillin-clavulanic acid; AMP, ampicillin; CAZ, ceftazidime; CIP, ciprofloxacin; CPD, cefpodoxime; CTX, cefotaxime; GEN, gentamicin; IPM, imipenem; MEM, meropenem; NIT, nitrofurantoin; SXT, trimethoprim-sulfamethoxazole; TGC, tigecycline; TZP, piperacillin-tazobactam.

### AMR trends of GPB causing UTIs

Overall, the percentage of resistance of GPB increased to some antibiotics (trimethoprim-sulfamethoxazole, erythromycin, tetracycline, levofloxacin, and fosfomycin) and decreased to others (penicillin and gentamicin). However, the differences were not significant. Interestingly, we observed no resistance of GPB toward linezolid, daptomycin, mupirocin, rifampicin, and vancomycin in both periods (Supplementary Table I). Due to the small numbers of GPB, the comparison between health care facilities was not reasonable.

### The trend of extended-spectrum β-lactamase–producing Enterobacterales causing UTIs

**Figure 3:**
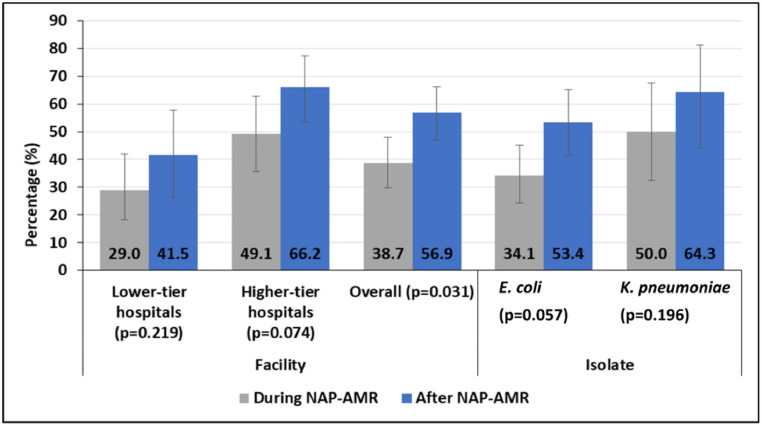
Proportions of ESBL-PE among E. coli and *K. pneumoniae* causing urinary tract infections. NAP-AMR, national action plan on antimicrobial resistance.

A total of 228 *Enterobacterales* isolates, consisting of *E. coli* (n = 162) and *K. pneumoniae* (n = 66), were tested for ESBL production. Of these, 108 (47.4%; 95% CI: 40.7-54.1%) were identified as ESBL-PE. The proportion of ESBL-PE increased significantly from 38.7% (46 of 119 [95% CI: 29.9-48.0%]) during NAP-AMR to 56.9% (62 of 109 [95% CI: 47.1-66.3%]) after NAP-AMR (*P* = 0.0307) (Figure 3).

### Factors associated with culture-positive UTIs caused by ESBL-PE

The univariate logistic regression analysis revealed that UTIs caused by ESBL-PE were significantly common among inpatients (57.1% vs 42.4%; odds ratio [OR] [95% CI]: 1.81 [1.04-3.15], *P* = 0.036), patients in higher-tier hospitals (58.4% vs 34.0%; OR [95% CI]: 2.73 [1.58-4.68], *P* <0.0001), and patients enrolled after NAP-AMR (56.1% vs 38.7%; OR [95% CI]: 2.09 [1.23-3.55], *P* = 0.006). Conversely, current antibiotic use during sample collection was found to be a protective factor against UTIs caused by ESBL-PE (33.3% vs 51.7%; OR [95% CI]: 0.47 [0.25-0.88], *P* = 0.019). However, the multivariate logistic regression analysis showed that UTIs caused by ESBL-PE remained significantly common in patients in higher-tier hospitals (58.4% vs 34.0%; OR [95% CI]: 2.51 [1.41-4.48], *P* = 0.002) (Supplementary Table 2).

## Discussion

The current study aimed to evaluate the epidemiology and antimicrobial resistance trends of pathogens causing UTIs during and after the implementation of the NAP-AMR 2017-2022 in Mwanza, Tanzania. The cumulative prevalence of culture-positive UTIs in both periods was 22.8%, which is in line with previous studies among HIV-positive pregnant women (21.4%) in 2017 [15], pregnant women (28.0%) in 2019 [16], and outpatients (26.5%) in 2022 [6] in the same region. Our observations indicate that UTIs are still a health care concern in our region. Although there was a slight decrease in UTIs in high-tier hospitals, there was also slight increase in UTIs in the lower-tier hospitals when comparing the two periods. The changing trends of UTIs in higher-tier and lower-tier hospitals may be attributed to various factors such as variations in patient demographics, such as patients’ age (23 [5-40] vs 29 [8-44] years, *P* <0.0001), clinical characteristics, and health care practices [17,18]. Patients in the higher-tier hospitals are more likely to be exposed to antibiotics in the cascade of referral systems and hence, have fewer positive urine cultures but more resistant isolates.

It is worth noting that the proportion of patients using antibiotics at the time of enrollment decreased from 47.1% during NAP-AMR to 0.3% after NAP-AMR (*P* = 0.0526). This observation could be attributed to the fact that the second half of the first cohort was done amid COVID-19 pandemic, which may have resulted in an increase in antibiotic use in health care facilities and the community for prevention and treatment of symptoms related to COVID-19. This differs from the second cohort, which took place after the pandemic and most of its participants were enrolled as outpatients, unlike those enrolled during NAP-AMR. After the pandemic, the high demand for antibiotic use (due to COVID-19) in the general community was absent. In addition, it is well noted that inpatients are more likely to be exposed to antibiotics than outpatients. However, almost the same proportion of participants enrolled during and after NAP-AMR had previously used antibiotics within 3 months before enrolling in the current study.

GNB remained the predominant causative agents of UTIs in both periods. However, their proportion decreased significantly (from 88.1% to 74.3%, *P* <0.0001). This shift may be attributed by increased antibiotics use during NAP-AMR from COVID-19 pandemic, which may have suppressed wild-type GNB, resulting in the emergence of resistant GPB. For instance, we observed an increased proportions of *E. faecalis* and *E. faecium,* which are previously reported to be intrinsically resistant toward multiple antibiotics [19]. Similar to previous studies in the same settings [6,15,16] and elsewhere [20], *E. coli* remained the most prevalent agent of UTIs, highlighting its persistence as a major pathogen, followed by *K. pneumoniae* [15,16]. The predominance of these two pathogens is not surprising because these are gut microbiota which can easily contaminate perineum and genital regions, resulting into ascending infections in the urinary tract mostly among women.

The detection of rare bacteria species such as *Citrobacter braakii, Pseudomonas putida, Proteus terrae, Acinetobacter haemolyticus, Acinetobacter schindleri, Enterococcus avium, Empedobacter brevis, Leclercia adecarboxylata, Paenibacillus alvei*, and *Streptococcus vestibularis* is largely linked to the matrix-assisted laser desorption ionization-time of flight Vitek MS advanced identification technology used in this study. These species were isolated exclusively after NAP-AMR, suggesting that these bacteria are more resilient to the changes in antibiotic usage patterns or have become more pathogenic due to the removal of competitive strains during the NAP-AMR period. In addition, these rare bacterial species have been reported as emerging human pathogens [21], harboring virulence genes [13] and intrinsic antibiotic resistance [22], and are associated with multidrug-resistance (MDR) and extensive drug-resistance infections [23,24]. For instance, the role of CoNS in UTIs, along with their virulence genes and AMR genes, has been previously documented in the same context [13]. In addition, *P. alvei* was reported to cause UTI [25], *A. calcoaceticus* was reported to cause bloodstream infection [24], *P. putida* was reported to cause UTI and bloodstream infection [23], *L. adecarboxylata* was reported to cause UTI and cellulitis [21], and *R. stabekisii* was reported to cause dental root canal infection [26].

AST showed that there has been a significant increase in antibiotic resistance toward cefpodoxime, ceftazidime, and ciprofloxacin among GNB. Similar findings were reported elsewhere, particularly, after COVID-19, associated with increased antibiotic use [27]. Remarkably, nitrofurantoin has demonstrated its continued efficacy as an antibiotic for treating uncomplicated UTIs in lower- and higher-tier hospital settings, as recommended by the Tanzanian standard treatment guidelines [9]. The most notable finding in our study is the significant increase in the proportion of ESBL-PE (38.7% vs 56.9%, *P* = 0.0307). This observation may imply the impact of increased use of antibiotics during the COVID-19 pandemic [8]. The increase in ESBL-PE underscores the urgency of addressing the AMR crisis and the need for comprehensive AMS programs in our setting. Similar to a previous UTIs study in the cascade of referral health care systems [28], the resistance trend in higher-tier hospitals is more critical than in lower-tier hospitals, suggesting that lower-tier hospitals use less antibiotics and, therefore, have less resistance or have a different profile of UTI-causing GNB. These findings suggest that higher-tier hospitals face more severe antibiotic resistance challenges, attributed by patient load, more complex and severe cases, and excessive use of broad-spectrum antibiotics, which exert greater selective pressure [29].

On the other hand, GPB increased high resistance toward trimethoprim-sulfamethoxazole, erythromycin, tetracycline, levofloxacin, and fosfomycin. Interestingly, we observed no resistance of GPB toward linezolid, daptomycin, mupirocin, rifampicin, and vancomycin in both periods, which is an appealing finding because these are last-resort antibiotics for GPB. Similar findings were reported previously [15,16]. However, it is essential to remain vigilant in monitoring the resistance patterns for these antibiotics because misuse or overuse can lead to emerging resistance in the future [29].

Moreover, we observed that UTIs caused by ESBL-PE were significantly common among inpatients, patients in higher-tier hospitals, and patients enrolled after NAP-AMR. Inpatients are more likely to be infected by MDR bacteria due to high exposure to antibiotics and reservoirs (*viz.* hospital environment) of MDR bacteria. Frequent exposure to antibiotics enhances selective pressure on the normal microbiota, leading to the proliferation of MDR bacteria, resulting in subsequent endogenous MDR infections [29]. In addition, higher-tier hospitals are linked with infections by MDR bacteria because they are often dealing with complex cases and use a broad range of antibiotics, which may contribute to the increased prevalence of ESBL-PE-associated UTIs. This finding emphasizes the urgent need for stringent AMS programs and infection control measures within higher-tier hospitals to curb the rise of resistant strains. Furthermore, the increased proportion of UTIs caused by ESBL-PE after NAP-AMR can be linked to several factors associated with the COVID-19 pandemic in 2020 and 2021: a significant rise in the use of antibiotics, disinfectants, and antiseptics in the community and health care settings; compromised AMS programs and infection prevention and control measures; and changes in microbial ecology due to the extensive and prolonged use of broad-spectrum antibiotics [8,30].

Due to budget constraints, the data collection for the second cohort was shorter than the first cohort. However, we addressed issues raised and observed during the first cohort. Therefore, in the second cohort, we made significant improvements by disseminating the research findings of the first cohort, sensitizing doctors to utilize microbiology laboratories, and re-training staff to improve case screening and laboratory diagnosis. This was particularly crucial in district hospitals (lower-tier hospitals) where culture and AST were initially lacking. As a result, the total number of study participants in the second cohort was relatively similar to the first cohort, despite being conducted in a relatively shorter period.

### Study limitations

The limited number of GPB in this study has limited plausible extrapolation of the resistant trends in this group of pathogens, underscoring a need for long-term AMR surveillance targeting GPB.

## Conclusion

The implementation of the NAP-AMR (2017-2022) encountered significant challenges exacerbated by heightened antibiotic use during the COVID-19 pandemic, particularly, in 2020 and 2021. This increase in antibiotic consumption raised concerns about the acceleration of AMR, undermining the efforts of the NAP-AMR in combating this pressing public health concern. Our study shows increasing AMR from lower- to higher-tier hospitals and after the implementation of NAP-AMR, notably, in third-generation cephalosporins and ciprofloxacin. However, nitrofurantoin has maintained its effectiveness in treating uncomplicated UTIs in lower- and higher-tier hospital settings. Furthermore, the lower resistance to last-resort antibiotics in higher-tier hospitals is promising but necessitates strengthening AMS programs to prolong their efficacy. In addition, future endeavors should focus on characterizing predominant UTI pathogens to better understand intra- and inter-hospital transmission dynamics, thereby guiding effective infection prevention and control measures.

## Declarations of competing interest

The authors have no competing interests to declare.

## Appendix Supplementary materials

Image, application 1

Image, application 2
