# Supplementary material for: Epidemiology and antimicrobial resistance trends of pathogens causing urinary tract infections in Mwanza, Tanzania: A comparative study during and after the implementation of the National Action Plan on Antimicrobial Resistance (2017-2022)
Source: Int J Infect Dis. 2024 Oct;147:None. doi: 10.1016/j.ijid.2024.107208 (PMC11442316; doi:10.1016/j.ijid.2024.107208)
Supplement: Supplementary file 1 [file mmc1.docx]

**Supplementary Table 1: The comparison of percentage resistance of Gram-positive bacteria causing urinary tract infections among patients with the clinical diagnosis of urinary tract infections during and after National Action Plan on Antimicrobial Resistance**

| Antibiotic agent tested | During NAP-AMR (N = 10 – 31) | | After NAP-AMR (N = 26 – 65) | | P value |
| --- | --- | --- | --- | --- | --- |
|  | **% resistance** | **95%CI** | **% resistance** | **95%CI** |  |
| PEN | 77.8 | 39.9 – 97.2 | 40.0 | 5.3 – 85.3 | 0.1531 |
| SXT | 60.0 | 26.2 – 87.8 | 66.7 | 43.0 – 85.4 | 0.3869 |
| ERY | 50.0 | 18.7 – 81.3 | 71.4 | 47.8 – 88.7 | 0.1908 |
| GEN | 50.0 | 18.7 – 81.3 | 38.9 | 17.3 – 64.3 | 0.3511 |
| TCY | 40.0 | 12.2 – 73.7 | 57.1 | 34.0 – 78.2 | 0.2765 |
| LVX | 25.9 | 11.1 – 46.3 | 52.8 | 38.6 – 66.7 | 0.1012 |
| FOS | 10.0 | 0.2 – 44.5 | 44.4 | 21.5 – 69.2 | 0.2545 |
| CLI | 0.0 | 0.0 – 30.8 | 5.0 | 0.1 – 24.8 | - |
| LNZ | 0.0 | 0.0 – 12.8 | 0.0 | 0.0 – 6.6 | - |
| DAP | 0.0 | 0.0 – 30.8 | 0.0 | 0.0 – 18.5 | - |
| NIT | 0.0 | 0.0 – 45.9 | 11.8 | 1.5 – 36.4 | - |
| FUS | 0.0 | 0.0 – 30.8 | 27.8 | 9.7 – 53.5 | - |
| MUP | 0.0 | 0.0 – 36.9 | 0.0 | 0.0 – 60.2 | - |
| RIF | 0.0 | 0.0 – 33.6 | 0.0 | 0.0 – 18.5 | - |
| TGC | 0.0 | 0.0 – 13.2 | 10.6 | 3.5 – 23.1 | - |
| TEC | 0.0 | 0.0 – 13.2 | 9.4 | 3.1 – 20.6 | - |
| VA | 0.0 | 0.0 – 12.8 | 0.0 | 0.0 – 6.7 | - |

Key: PEN=penicillin, SXT=trimethoprim-sulfamethoxazole, ERY=erythromycin, LVX=levofloxacin, TCY=tetracycline, GEN=gentamicin, CLI=clindamycin, LNZ=linezolid, DAP=daptomycin, FOS=fosfomycin, FUS=fusidic acid, MUP=mupirocin, RIF=rifampicin, TGC=tigecycline, TEC=teicoplanin, and VA=vancomycin.
