# Supplementary material for: Epidemiology and antimicrobial resistance trends of pathogens causing urinary tract infections in Mwanza, Tanzania: A comparative study during and after the implementation of the National Action Plan on Antimicrobial Resistance (2017-2022)
Source: Int J Infect Dis. 2024 Oct;147:None. doi: 10.1016/j.ijid.2024.107208 (PMC11442316; doi:10.1016/j.ijid.2024.107208)
Supplement: Supplementary file 2 [file mmc2.docx]

**Supplementary Table 2: Factors associated with culture positive urinary tract infections by extended-spectrum beta-lactamase-producing Enterobacterales among patients with the clinical diagnosis of urinary tract infections in Mwanza, Tanzania**

| Independent variables | | ESBL-PE UTIs | | Statistical tests | | | | | |
| --- | --- | --- | --- | --- | --- | --- | --- | --- | --- |
|  |  | **Negative**  **(N=120)** | **Positive**  **(N=108)** | **Chi-square analysis** | | **Univariate analysis** | | **Multivariate analysis** | |
|  |  | **n(%)** | **n(%)** | **X^2^** | **P value** | **OR[95%CI]** | **P value** | **OR[95%CI]** | **P value** |
| Age in years | | 30 [6 – 47] | 33 [6 – 44] | - | 0.7018 |  |  |  |  |
| Sex | Female | 84 (53.1) | 74 (46.9) | 0.0586 | 0.809 | 1 | 0.809 |  |  |
|  | Male | 36 (51.4) | 34 (48.6) |  |  | 1.07[0.61 – 1.88] |  |  |  |
| Residency | Rural | 61 (51.7) | 57 (48.3) | 0.0861 | 0.769 | 1 | 0.769 |  |  |
|  | Urban | 59 (53.6) | 51 (46.4) |  |  | 0.92[0.55 – 1.55] |  |  |  |
| Patient category | Outpatient | 87 (57.6) | 64 (42.4) | 4.4555 | 0.035 | 1 | 0.036 | 1 | 0.194 |
|  | Inpatient | 33 (42.9) | 44 (57.1) |  |  | 1.81[1.04 – 3.15] |  | 1.49[0.82 – 2.72] |  |
| Hospital level | Lower-tier | 68 (66.0) | 35 (34.0) | 13.5067 | <0.0001 | 1 | <0.0001 | 1 | 0.002 |
|  | Higher-tier | 52 (41.6) | 73 (58.4) |  |  | 2.73[1.58 – 4.68] |  | 2.51[1.41 – 4.48] |  |
| History of fever | No | 71 (56.8) | 54 (43.2) | 1.9285 | 0.165 | 1 | 0.166 |  |  |
|  | Yes | 49 (47.6) | 54 (52.4) |  |  | 1.45[0.86 – 2.45] |  |  |  |
| History of antibiotic | No | 84 (54.5) | 70 (45.5) | 0.6971 | 0.404 | 1 | 0.404 |  |  |
|  | Yes | 36 (48.6) | 38 (51.4) |  |  | 1.27[0.73 – 2.21] |  |  |  |
| Current on antibiotic | No | 84 (48.3) | 90 (51.7) | 5.5908 | 0.018 | 1 | 0.019 | 1 | 0.073 |
|  | Yes | 36 (66.7) | 18 (33.3) |  |  | 0.47[0.25 – 0.88] |  | 0.49[0.23 – 1.07] |  |
| History of admission | No | 107 (51.7) | 100 (48.3) | 0.7978 | 0.372 | 1 | 0.374 |  |  |
|  | Yes | 13 (61.9) | 8 (38.1) |  |  | 0.66[0.26 – 1.65] |  |  |  |
| Chronic disease | No | 102 (51.0) | 98 (49.0) | 1.7390 | 0.187 | 1 | 0.191 |  |  |
|  | Yes | 18 (64.3) | 10 (35.7) |  |  | 0.58[0.25 – 1.31] |  |  |  |
| Study duration | During NAP-AMR | 73 (61.3) | 46 (38.7) | 7.5797 | 0.006 | 1 | 0.006 | 1 | 0.274 |
|  | After NAP-AMR | 29 (43.9) | 37 (56.1) |  |  | 2.09[1.23 – 3.55] |  | 1.42[0.76 – 2.66] |  |
| Organism | *E. coli* | 91 (56.2) | 71 (43.8) | 2.8151 | 0.093 | 1 | 0.095 |  |  |
|  | *K. pneumoniae* | 29 (43.9) | 37 (56.1) |  |  | 1.64[0.92 – 2.91] |  |  |  |
